# Advances in Molecular Dynamics Simulations and Enhanced Sampling Methods for the Study of Protein Systems

**DOI:** 10.3390/ijms21176339

**Published:** 2020-09-01

**Authors:** Raudah Lazim, Donghyuk Suh, Sun Choi

**Affiliations:** College of Pharmacy and Graduate School of Pharmaceutical Sciences, Ewha Womans University, Seoul 03760, Korea; raudah@ewha.ac.kr (R.L.); dsuh@ewha.ac.kr (D.S.)

**Keywords:** molecular dynamics simulation, enhanced sampling, protein-protein interactions, binding free energy, protein-ligand binding affinity

## Abstract

Molecular dynamics (MD) simulation is a rigorous theoretical tool that when used efficiently could provide reliable answers to questions pertaining to the structure-function relationship of proteins. Data collated from protein dynamics can be translated into useful statistics that can be exploited to sieve thermodynamics and kinetics crucial for the elucidation of mechanisms responsible for the modulation of biological processes such as protein-ligand binding and protein-protein association. Continuous modernization of simulation tools enables accurate prediction and characterization of the aforementioned mechanisms and these qualities are highly beneficial for the expedition of drug development when effectively applied to structure-based drug design (SBDD). In this review, current all-atom MD simulation methods, with focus on enhanced sampling techniques, utilized to examine protein structure, dynamics, and functions are discussed. This review will pivot around computer calculations of protein-ligand and protein-protein systems with applications to SBDD. In addition, we will also be highlighting limitations faced by current simulation tools as well as the improvements that have been made to ameliorate their efficiency.

## 1. Introduction

Proteins are vital constituents of living organisms, responsible for myriads of life-sustaining cellular processes such as molecular recognition, signal transduction, protein localization, and enzyme catalysis. These biological events are navigated by protein motions as well as physical interactions established between proteins and partnering molecules such as ligands, peptides, proteins and nucleic acids [1]. Progresses made in structural biology and biophysical characterization methods (X-ray crystallography, nuclear magnetic resonance (NMR) and cryo-electron microscopy) have resulted in the exponential growth in the number of three-dimensional structures available for proteins, protein-ligand and protein-protein complexes. This encourages the study of protein dynamics at the atomic level which is made facile with the advent of molecular dynamics (MD) simulation.

The introduction of MD simulation to molecular biology has enabled researchers to access a microscopic visualization of biological processes. While the history of MD simulation of macromolecules began with a simple structural investigation of a small protein, namely bovine pancreatic trypsin inhibitor, the field has matured to a stage that calls for the development of new technology to facilitate the study of larger proteins and protein complexes with more intricate dynamics [2,3,4]. MD simulations conducted on a variety of protein systems—single proteins, protein-ligand and protein complexes—have provided valuable insights on protein stability, protein-ligand binding and protein-protein association, among others. These applications when assimilated into the drug discovery pipeline will empower researchers to identify crucial interactions necessary for the favorable binding of small molecules, peptides, and proteins to binding pockets and/or protein-protein interfaces. The synergistic use of MD simulations and free energy calculations will further aid in harnessing thermodynamic and kinetic parameters essential for the prediction of binding free energies. The abilities of these computational tools to determine binding interactions and binding affinities are beneficial for structure-based drug design (SBDD) as such predictions could be utilized to accelerate drug development by aiding hit-to-lead optimization to afford drugs with enhanced specificity and selectivity.

**Figure 1:**
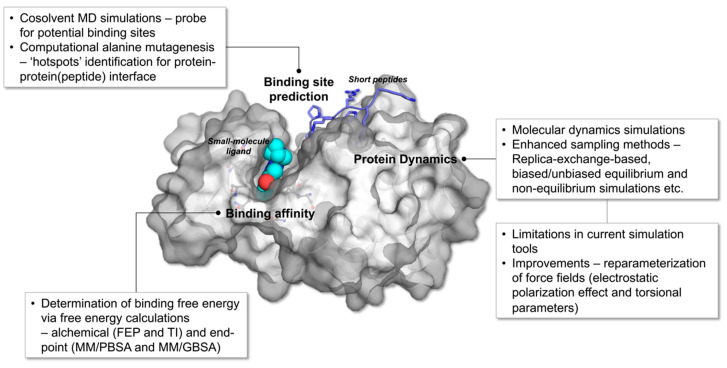
Overview of MD simulation and enhanced sampling methods utilized in the study of protein-protein (peptide) and protein-ligand complexes. The figure is constructed using the crystal structure of human PIM1 kinase in complexed with imidazopyridazin inhibitor and substrate peptide (PDB id: 2C3I).

The advancement in computing architecture and algorithms has spurred the extensive growth of MD simulation tools over recent decades. Architectural developments of computers resulted in the invention of graphics processing units (GPUs) and parallel computing while the refinement of algorithms leads to a more accurate rendering of the potential energy surface (PES) of the system and better conformational sampling. The combination of both facets of computing has enabled a more reliable prediction of the physical properties of proteins as well as the observation of biologically relevant protein structures at the atomic level, a feat that requires timescales beyond milliseconds, expanded length scales, and enhanced sampling techniques that facilitate high free energy barrier crossing [5]. The burgeoning use of MD simulation as a main tool in the computational studies of proteins as well as a complementary machinery in experimental studies has propelled actions to improve various aspects of MD simulations, warranting better predictive power and more reliable in silico analysis of protein structures, dynamics and functions. In this review, we will provide an overview of simulation strategies, mainly all-atom MD simulation and enhanced sampling methods, used to examine protein structures and dynamics to facilitate the understanding of mechanisms regulating biological processes namely, protein-ligand binding and protein-protein association. The capitalization of the predictive power of MD simulation and enhanced sampling methods to determine binding free energies as well as potential binding sites are also discussed. Furthermore, limitations of current simulation techniques as well as improvements made to optimize the accuracy of MD simulation/enhanced sampling methods are highlighted. Figure 1 reflects a summary of the topics discussed in this review.

## 2. Computational Methods for the Conformational Study of Protein-Protein & Protein-Ligand Systems

### 2.1. Enhanced Sampling Methods Used in Protein-Related Studies

MD simulation based on classical mechanics has been widely used for sampling conformational space of biomolecules, ranging from small molecules [6], peptides [7], to large proteins [8]. To observe important biological activities, such as enzymatic catalysis or protein-protein association/dissociation, one might need enhanced sampling methods rather than straight unbiased MD due to the rugged free energy surface of complex biological molecules and their extensive timescale of milliseconds to microseconds. Various methods have been proposed to overcome this shortcomings using multiple replicas of the same system [9,10,11,12,13,14], biasing the underlying potential energy function [15,16,17,18,19,20,21], using models that allow greater time-steps [22], and generating kinetic models followed by adaptive sampling [23,24].

**Figure 2:**
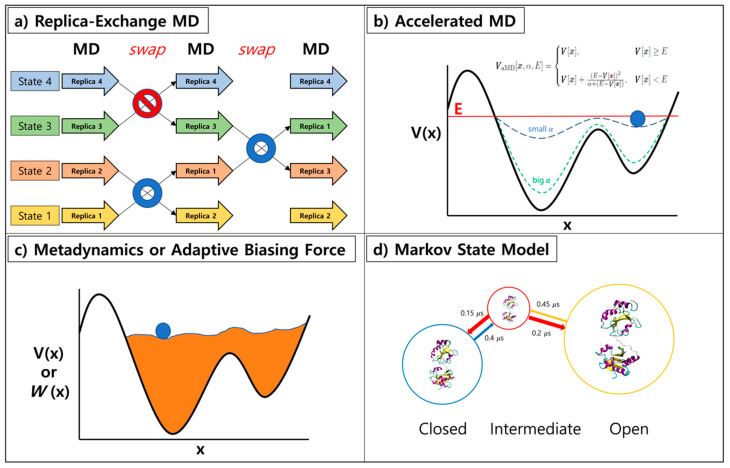
Overview of enhanced sampling methods: (**a**) replica-exchange MD simulation, (**b**) accelerated MD, (**c**) metadynamics or adaptive biasing force, and (**d**) Markov state model.

Replica-exchange molecular dynamics (REMD) method has been suggested by Sugita and Okamoto [9]. Multiple copies of the system with different temperatures or Hamiltonian are run concurrently and exchanged at fixed interval in this generalized ensemble methodology (Figure 2a). The exchanges are permitted based on the Metropolis criterion, generating a generalized ensemble [25]. Since swapping with neighboring replicas of different temperature or Hamiltonian can enhance the convergence of MD simulation, REMD has been extensively adopted in various ensemble simulations. One could apply this method to many expanded ensembles when proper exchange acceptance criterions are used [26]. The applications of REMD are as follows: replica-exchange umbrella sampling (REUS) [10], temperature REMD [11,12], Hamiltonian REMD [13], free energy perturbation with REMD (FEP/REMD) [27], and replica-exchange constant-pH [14,28,29].

Hydrogen mass repartitioning (HMR) allows increment of intrinsic time-step from 2 fs to 4 fs for solvated biomolecular systems. The time-step of normal MD is determined by the fastest motions in the system, which is normally a vibrational motion of hydrogen bond, limited to 2 fs for a stable simulation. The 4 fs time-step, a factor of 2 speed up, is granted by increasing the mass of hydrogen to about 3 amu and decreasing the equivalent mass from connected heavy atom. Hopkins et al. tested HMR on various benchmark systems, ranging from 3-residue peptide to 129-residue protein, hen egg white lysozyme (HEWL), and concluded there is no significant difference in kinetics and thermodynamics when HMR is used [30]. Balusek et al. assessed the validity of HMR for the membrane simulation, and figured out most structural properties (area-per-lipid, electron density profile, order parameters) being identical, but some kinetic properties such as diffusion constant showed some differences [31].

One can enhance the sampling by running simulations on modified potential energy functions, boosted and smoothened ones. Accelerated Molecular Dynamics (aMD) suggested by McCammon [16] directly modifies the potential energy function to expedite the transition of molecule from the local minimum. Simulating proteins and polypeptide are often accompanied with high potential energy barrier in between distinguished states of the system. When aMD is applied to the system, a robust bias energy is added to the potential energy function according to the set of variables (a threshold energy (E) and a boosting constant (α) to reduce the potential energy barrier (Figure 2b). Smoothened potential energy function of a system allows its exchange rate between various conformational states. Similar type of approaches with different rules for modification to the potential energy functions exist, namely local elevation and conformational flooding [32,33]. For methods above, reweighting of the perturbed free energy surface allows one to recover the original Boltzmann distribution of the system. However, proper selection of the boost potential is of importance as overestimation could invalidate events observed during the trajectory [34]. Nonetheless, usage of this algorithm has been successful on rare-event observations of many complex biological systems [35,36,37].

Another type of enhanced sampling method embraces continuous modifications of the energy profile during the simulation. Metadynamics (MTD) suggested by Parinello’s group modifies potential energy function in a regular basis during the simulation. Addition of potential energy to visited states discourages repeated sampling of these states, hence promoting the inspection of unexplored conformational space (Figure 2c). Adaptive biasing force (ABF) method memorizes the applied force at each point during the simulation and accumulates these forces in bins along the transition coordinate to calculate the average force at each coordinate interval [38]. Flattening of the free energy landscape was subsequently performed by utilizing the potential derived from integrating the average force applied. Both methods are successful when few meaningful collective variables and proper transition coordinate are chosen. Recent work by Blanc et al. highlights the recovery stroke of myosin VI using ABF [39] and the folding mechanism of protein G from streptococcal bacteria has been studied by Granata et al. with metadynamics [40].

The Markov state model (MSM) is a different type of approach, generating a kinetic model from a long unbiased MD trajectory [23,41]. MSM is constructed by building microstates from the initial trajectory, then a transition matrix is calculated from the states with a carefully chosen lag time used to define transition (Figure 2d). The dimension reduction is often performed with principle component analysis (PCA) [42] or time-structure independent component analysis (tICA) [43]. PyeMMA by Noe’s group [44] and MSMbuilder by Pande’s group [45] are two mainstream open sources for MSM construction. Using MSM, the largely obscure multi-dimensional data is turned into a simplified model readily understood by human beings, and one can utilize this for adaptive sampling. The nature of protein/peptide folding has been readily studied by this approach [46]. MSM can be used to direct sampling to less explored regions, allowing users to run more simulations at these points resulting in an improved model of the system of interest [47].

### 2.2. Recent Applications of Enhanced Sampling Methods for Protein Complexes

Protein-protein association plays a pivotal role in a myriad of cellular signaling pathways. Misguided protein-protein interactions (PPIs) have been linked to neurodegenerative diseases such as Alzheimer’s diseases and Parkinson’s disease, and the inhibition of protein dimerization/oligomerization has been proposed as a potential therapeutic treatment for cancer [48,49,50]. The biological significance of PPIs is further reflected by the increasing number of peptide-based drugs made available over the years, prompting the need to understand the mechanism governing the binding of peptide ligands to proteins. Numerous computational studies conducted to comprehend protein (peptide)-protein interactions have been conducted and oftentimes, these studies extended beyond traditional MD simulations. Wang et al., in order to precisely model protein-peptide binding, utilized the fast Fourier transform (FFT) based docking method, ClusPro PeptiDock, for the docking of peptide ligands, and refined the resulting protein-peptide complexes using Gaussian aMD (GaMD) simulation [51,52]. The GaMD simulation is analogous to the aMD simulation (vide supra) in that both enable unconstrained enhanced sampling without the need of a predefined collective variable. However, during the GaMD simulation, a boost potential in the form of a Gaussian function is implemented, to ensure more reliable reweighting during the construction of the potential of mean force (PMF) profile [53]. Using the aforementioned *PeptiDock+GaMD* method, Wang et al. successfully predicted the binding poses of three distinct peptides to their partnering proteins, namely, SH3 domain, XLP protein and PIM1 kinase [52]. The resulting protein-peptide complexes concurred well with their respective X-ray crystal structures, with lowest backbone root-mean-square-deviations (rmsds) ranging from 0.6 to 2.7 Å. When refinement was not performed, the protein-peptide complexes acquired using just PeptiDock recorded lowest backbone rmsds in the range of 3.3 to 4.8 Å. The better backbone rmsds obtained when refinement of the protein-peptide complexes was conducted after docking demonstrated the importance of considering protein flexibility and conformational heterogeneity when predicting binding poses of protein-peptide complexes. The same could be extended to protein-ligand and protein-protein complexes.

Besides modelling protein-peptide binding, mechanistic studies delineating protein-protein association and dissociation at the molecular level are also necessary to obtain a comprehensive picture of how native protein-protein contacts are established. Pan et al. introduced a protocol involving the synergistic use of long-timescale MD simulations with their newly developed enhanced sampling method, termed “tempered binding”, to investigate reversible protein-protein association [54]. The “tempered binding simulation” is comparable to simulated tempering, a global optimization method that dynamically changes the temperature of the system following the generalized Metropolis criterion [54,55]. However, instead of temperature, “tempered binding” updates the Hamiltonian function of the system by scaling the strength of interatomic interactions, such as near electrostatic interactions between proteins and between proteins and solvent molecules, through periodic Monte Carlo (MC) moves [54]. The on-the-fly scaling improves sampling efficiency by preventing conformational trapping, thus inducing the dissociation of long-lived states much faster than conventional MD simulation could. This consequently enabled the observation of reversible protein-protein association as demonstrated by the spontaneous association and dissociation of five protein-protein complexes to and from their respective native conformations. Conventional MD simulations performed on the same protein-protein complexes revealed similar spontaneous protein-protein association as well. However, protein-protein dissociation was not observed once the native conformation of the complex was achieved and kinetic trapping of the complex in non-native conformation was also discerned, corroborating the benefits of enhanced sampling methods in the study of reversible protein-protein association.

Even though enhanced sampling methods advocate better sampling efficiency than conventional MD simulations in most cases, selecting the best enhanced sampling technique for your system of interest is also vital to ensure that a reliable free energy profile can be obtained. In a recently published study by Wingbermühle and Schäfer, a systematic comparison of the performance of four enhanced sampling techniques, namely, US, REUS, well-tempered MTD, replica exchange with solute tempering 2 (REST2), and biased exchange umbrella sampling (BEUS), was performed to examine the partial dissociation of the N-terminus of an antigenic peptide from Major Histocompatibility Complex class I (MHC I) protein [56]. Through this study, they concluded that BEUS afforded the most reliable PMF profile of the protein-peptide dissociation, given by the clear-cut segregation of the fully-bound and the partially dissociated states of the protein-peptide complex in the energy profile, as well as the consistent sampling of conformations along the reaction coordinates (RC). This study also offered useful insights, which could be applied when simulating biological systems that are conflicted in terms of enthalpic and entropic contribution. A comprehensive understanding of the system enables proper selection of RC or collective variables that permits the sampling of entropy-driven events, such as the partially dissociated state. While this could elevate the element of bias during the simulation, BEUS was suggested to have mitigated this effect by using MC updates of RC values, thus providing room for other degrees of freedom to fluctuate.

## 3. Computational Methods for the Prediction of Protein-Protein (Peptide) and Protein-Ligand Binding

### 3.1. Free Energy Calculations for Prediction of Protein-Ligand and Protein-Protein Binding Affinity

The prediction of protein-ligand binding affinity has been a main goal in drug discovery field since the magnitudes of the affinity and the selectivity of designed ligands/drugs are crucial in SBDD. Free energy perturbation (FEP) and thermodynamic integration (TI) methods have been suitable for this aspect as both provide a relatively reliable prediction of protein-ligand binding affinity. Both require MD simulations at different values of lambdas ranging from 0 to 1 which represent their perturbation state, 0 being the original state and 1 being the fully perturbed state. Then, applying either Zwanzig’s equation for FEP or thermodynamic integration formalism would disclose the associated free energy of perturbation [57,58]. Absolute binding free energy (ABFE) calculation, where total annihilation of the ligand in the binding pocket followed by its reappearance at bulk state where receptor is absent, is extensively used for small ligands. As free energy is a state function, the alchemical FEP route to getting binding free energy of ligand is as follows: (1) ‘locking the ligand’, restraining conformational, translational, and rotational degrees of freedom at bound state, (2) ‘disappearing the locked ligand’, turning off the interaction between ligand and its surroundings, (3) ‘translocating ligand’ of which corresponding free energy is zero, (4) ‘reappearance of the locked ligand’, turning on the interaction between ligand and its surroundings at bulk state, and (5) ‘unlocking the ligand’, releasing of the three restraints from (1). Aldeghi and co-workers performed ABFE simulations for a set of diverse inhibitors binding to bromodomain-containing protein 4 (BRD4) for actual pharmaceutical targets and drug-like molecules, and obtained a result giving excellent agreement with experimental data [59]. Lenselink et al. tested binding free energies of congeneric ligands to four different GPCRs using FEP+ from Schrödinger and obtained results in great agreement with experimental results [60,61,62].

This alchemical route can also be utilized when mutating a key residue in silico, obtaining the relative free energy difference upon mutation, ∆∆G [63]. Two types of topologies can be used for relative FEP where ‘disappearing certain group and reappearing counterpart’ takes place for “dual topology” [64], and ‘perturbation of an existing atom to different type of atom’ is done for “single topology” [65]. The difference between the two types of topologies is whether the starting and ending states share a perturbed atom. From methane to ethane perturbation, for example, dual topology will have another dummy methyl group attached to the carbon in methane. For single topology, one of the hydrogens in the methane becomes carbon while three hydrogens, non-interacting dummy atoms connected to the new carbon atom when system was methane, appear accordingly. Relative free energy calculations are cheaper than its absolute counterpart, of course, achieving convergence faster with fewer particles to be perturbed. Loeffler et al. studied the reproducibility of FEP among different MD packages to compare their reliability and to generate the benchmark protocol [66]. The results showed that the relative free energies calculated are equal to the difference in absolute free energies between the two states, hence proving the robustness of the methods and tools available in the field. Gapsys et al. successfully recovered the relative free energy from alchemically mutating residues [67]. The study was conducted towards 762 mutations and the changes in protein thermostability were predicted with remarkable accuracy.

To calculate protein-protein binding free energy, however, the alchemical FEP is not suitable because a simulation of ‘annihilating a protein’ from a solvated system is essentially impractical. If one can observe multiple associations/dissociations of protein complex with unbiased MD, constructing MSM upon the trajectory would be advantageous [68]. On the other hand, a PMF method suggested by Woo and Roux is suitable where dissociation is a rare-event due to the large affinity between proteins or kinetic trap [69]. Using this method, one can physically pull one protein apart from another during the simulation with six relative translational and orientational degrees of freedom defined. The user chooses arbitrarily three points in each protein, (P1, P2, and P3) and (L1, L2, and L3), in order to specify the spherical coordinates (r, θ, ϕ) = $\left(\bar{P1L1},\;∠P1L1L2,\;∠P1L1L2L3\right)$ and three Euler angles (Θ,Φ,Ψ) = (∠P2P1L1, ∠P2P1L1L2,∠P3P2P1L1) [70]. String method application to this method was recently published from the same group [71]. The method quickly searches for the minimum free energy path (MFEP) along the separation path with implicit solvent model, generating a string of optimal separation path based on 6-dimensional relative position and orientation as defined above. The string is refined a few times with explicit solvent model, and MD simulation along the optimal path converges much faster than straight separation.

While many replicas are required in between the perturbation states for FEP methods and along the separation path for PMF-based and string methods, the computationally cheaper way to estimate binding free energy would be the molecular mechanics combined with Poisson-Boltzmann or generalized Born and surface area solvation (MM/PBSA or MM/GBSA). With the relatively short amount of sampling, one can utilize snapshots from the trajectory to obtain the binding free energy that is estimated by getting the difference between the free energy of the complex and that of each body. The free energies are calculated as $G=E_{MM}+G_{solv}-TS$, where $E_{MM}$ stands for the bonded, electrostatic, and van der Waals energy terms from molecular mechanics, $G_{solv}$ stands for solvation free energy, and TS stands for temperature and entropy respectively. The solvation free energy consists of polar and non-polar, where polar solvation free energy can be estimated using either Poisson-Boltzmann or generalized Born theorem and non-polar solvation free energy is predicted from a linear relation to the solvent accessible surface area (SASA). MM/PBSA and MM/GBSA have been widely used for estimating binding free energies and showed definitively better results when compared to empirical attempts such as docking and virtual screening [62]. Though affordable, unaccounted conformation entropy and free energy of water molecules in binding sites sometimes resulted in poor estimation [72].

### 3.2. Recent Methods for the Prediction of Binding ‘Hotspots’ for Protein-Ligand and Protein-Protein Association

The prediction of residues crucial for the formation of favorable protein-ligand or protein-protein interactions is an asset in the field of rational drug design. These decisive sets of amino acids are called ‘hotspots’ and numerous studies with the objective of quantifying the contribution of residues towards binding affinity have been conducted to facilitate precise identification of ‘hotspot’ residues [73,74,75,76,77]. Experimentally, the determination of ‘hotspots’ proceeds through traditional methods such as alanine scanning mutagenesis, structure-activity relationship by NMR and multiple solvent crystal structures (MSCS) [78,79,80]. However, these experimental approaches are often time-consuming and labor-intensive. Hence, computational tools that alleviate the workload involved in mapping binding pockets and protein-protein interfaces are highly sought to expedite the process of developing ligand/drugs that preferentially bind at these ‘hotspots’.

The cosolvent or mixed-solvent MD simulation is one of the most widely used computational tool in SBDD for its accuracy in identifying ligand binding sites [73,81]. This method is the theoretical equivalent of the MSCS method in that it uses small organic probes to sweep protein surfaces to locate binding ‘hotspots’, which include orthosteric (active site), allosteric and cryptic pockets. Allosteric binding site define a binding pocket that is located away from the active site and can modulate protein activity through allostery. The process of allostery involves the propagation of signals from the allosteric pocket—initiated through ligand binding or mutation of residues—to the active site. This allosteric communication proceeds through a network of residue-residue interactions and/or structural fluctuations [82,83,84,85,86]. Numerous studies on the allosteric regulation of proteins have been conducted in recent years for its potential application towards the design of novel therapeutic ligands with minimal side effects, as allosteric sites, contrary to orthosteric binding sites, are less conserved and are topographically more varied [87,88]. These studies reflected the importance of considering structural dynamics to acquire a better understanding of allosteric communications [86,89,90]. However, before jumping into the allosteric modulation, it is imperative for researchers to identify and characterize accurately the potential allosteric sites that could be targeted for drug design. Cryptic sites, a subset of allosteric binding sites, are dynamic binding pockets which appearance are typically induced through the binding of orthosteric ligand that effectuates conformational changes via induced-fit mechanism, ligand-induced conformational state transitions or the simultaneous occurrence of both events [91,92,93]. In recent years, the SBDD of orthosteric ligands has been slightly upstaged by the design of ligands targeting allosteric and cryptic binding sites—potential druggable targets that are gaining attention for its plausible contribution to the development of ligands with better specificity and selectivity with attenuated side effects. This makes cosolvent MD simulation an ideal tool for SBDD as it enables the exploration of the overall druggable space of proteins and could be a solution for therapeutically attractive proteins that previously were deemed ‘undruggable’ targets [94].

As the name suggests, cosolvent MD simulation uses solvent mixtures that usually consist of organic molecules or fragments that possessed key pharmacophore features crucial for ligand binding such as hydrophobic centers, aromatic rings, hydrogen bond donors and hydrogen bond acceptors [81]. There are various successful protocols utilizing cosolvent MD simulation for binding ‘hotspot’ prediction, namely, SWISH, MixMD, MDMix, SILCS, mCSM and many others [93,95,96,97,98,99,100,101]. A recent development of SWISH—a Hamiltonian replica-exchange-based method developed to survey hydrophobic patches on proteins for druggability—demonstrated proficiency of using cosolvent probe in precisely identifying cryptic pockets [93,95]. The SWISH with probe approach successfully uncovered the cryptic pocket of the Niemann-Pick Type C2 protein, which was alluded to be challenging as the cryptic pocket is flanked by β-sheets and is highly specific towards sterol-like molecules. The use of SWISH successfully prompted the opening of the cryptic site, allowing the binding of benzene probes (analogous to a hydrophobic/aromatic ring pharmacophore feature) which further exposed the cryptic pocket while simultaneously stabilizing its opened state [95].

CrypticScout, accessible through PlayMolecule platform (www.playmolecule.org), formulated a similar protocol, which engages benzene probes to facilitate the identification of cryptic pockets [91,101,102]. The cryptic sites, after the simulation, were characterized through the solvent accessible surface area (SASA) calculation, ‘hotspot’ overlaps (with reference to apo structure) and scoring functions defined by free energy grids and occupancy. In addition to benzene probes, there are also other organic probes available through CrypticScout, namely, acetone, imidazole, isopropanol, and phenol, a good representation of pharmacophore features (vide supra) important for ligand binding [101]. The development of analytical tools that aid in the analysis of cosolvent MD simulation, such as the cosolvent analysis toolkit [73], further engendered the accessibility of cosolvent MD simulation to both expert and non-experts alike, expediting the exploration of protein’s chemical space for drug design. These studies highlighted the importance of computer simulations to continuously evolve to meet current needs in protein research. MD simulations and enhanced sampling methods are highly productive for studies related to protein folding, conformational change, protein-ligand binding, PPIs and enzymatic reactions; whereby efficient conformational sampling is of the essence. However, in situations such as the discovery of cryptic pockets (vide supra), enhanced sampling may not be sufficient for the unravelling of these obscured binding sites. Instead, augmentation of MD simulations/enhanced sampling methods with organic fragment probes may be necessary to maneuver the exposure of these cryptic binding sites [93,95,101].

Alanine scanning mutagenesis is another experimental technique that has been computerized to accelerate ‘hotspot’ prediction, mostly at protein-protein interface. This method involved the site-directed mutagenesis of specific residues to alanine to assess the contribution of the amino acid towards protein stability and function [103,104,105]. Computational alanine scanning (CAS), a robust alternative to its experimental counterpart, permits the rapid scanning of protein surface for amino acid clusters vital for protein-protein association [74]. Over the years, several CAS methods have been developed, centering around free energy calculations performed on single structures or structure ensembles from NMR or MD trajectories to predict ‘hotspots’ at protein-protein interface [106,107]. PPIs mostly occur at large, shallow regions on the protein surface (Figure 1) and the interactions established are usually transient, making it difficult to distinguish protein-protein binding interface. Therefore, it is crucial to consider protein dynamics to determine residues that contribute significantly towards binding free energies. For instance, the flex ddG method in Rosetta utilized a synergy of global optimization, Monte Carlo sampling with ‘backrub’ implementation, and advanced energy functions to predict changes in the binding free energy upon mutation (ΔΔ*G*) [108]. Rosetta ‘backrub’ simulation accounts for the conformational flexibility of proteins through the sampling of backbone and side chain rotamers. This method has been demonstrated to reproduce localized structural heterogeneity observed experimentally via high-resolution crystal structures and NMR structures. The utilization of the ‘backrub’ method in flex ddG to sample degrees of freedom at the mutation site and the neighboring residues permitted accurate depiction of protein flexibility in nature, which translates to better estimation of ΔΔ*G* [109].

Another recently established CAS tool is the BUDE Alanine Scanning (BudeAlaScan) method, which was built upon the empirical free energy approach of a molecular docking algorithm, BUDE (Bristol University Docking Engine) [74,110]. Similar to flex ddG, this method considers conformational plasticity by considering a set of side chain rotamers for charged residues (Asp, Glu, Arg, Lys and His) to approximate the entropy loss resulting from the formation of inter-protein salt bridges between the side chains of the aforementioned charged residues and a fixed protein backbone. Ibarra et al. in introducing the BudeAlaScan method has also simultaneously compared the performance of several CAS methods, including flex ddG, in accurately predicting ‘hotspot’ residues. The comparable results achieved for flex ddG and BudeAlaScan emphasized the importance of considering conformational heterogeneity to obtain a more precise prediction of ΔΔ*G* [74]. This comparative study also observed quality ΔΔ*G* prediction from mutation Cutoff Scanning Matrix (mCSM), a machine learning approach that was trained based on several curated databases documenting changes in protein stability and protein-protein affinity upon mutation, such as SKEMPI and ProTherm [111,112,113,114]. The mCSM method uses structural graph-based signatures to represent the changes in the protein environment upon mutation. The graph-based signature denotes atoms as nodes and interatomic interactions as edges and encodes changes in the physicochemical properties of residues through the difference in pharmacophore count between wild type (WT) and mutated proteins. In the recently refurbished mCSM method, called mCSM-PPI2, an additional six features were utilized to train and test the prediction model namely: (i) the environment of the WT residues such as solvent accessibility, torsional fluctuations, depth of residue, and amino acid content in the neighborhood of the mutated residue, (ii) detection of mutation from or to glycine (flexible side chain) or proline (rigid side chain), (iii) evolutionary conservation, (iv) non-covalent interaction network analysis, (v) energy terms (interaction energies between protein chains and changes in the predicted folding free energy on mutation) and, (vi) atomic fluctuations [112]. All of these features aid in improving the predictive power of mCSM by considering protein flexibility and environment effect, both of which undergo considerable changes when mutations are introduced.

Besides the utilization of CAS in determining ‘hotspots’ at protein-protein interface, CAS has also been adapted for protein-ligand systems [76,77,115]. Liu et al. presents an efficient protocol that combines MM/GBSA and the interaction entropy (IE) approach developed by Duan et al. to calculate the contribution of specific residues to the overall protein-ligand binding free energy [77,115]. Typically, the inclusion of entropic contribution in end-point free energy calculation such as MM/GBSA and MM/PBSA involved the use of expensive normal mode analysis (NMA). However, the use of NMA in CAS will incur a huge computational cost and therefore is not practical for the assessment of ΔΔ*G* especially when a sizable mutation scan is required. The introduction of the IE approach offers a more rigorous and less expensive computation of binding entropy as it explicitly calculates the entropic contribution by directly observing the fluctuation in interaction energy between individual residues and ligand from a single MD simulation, hence eliminating the additional computational cost required for entropy calculation [115]. The implementation of this CAS approach in the investigation of the binding of three drugs to anaplastic lymphoma kinase (ALK) and its mutants revealed ‘hotspot’ residues that were critical for protein-ligand binding. Considerable agreement between the calculated and the experimentally reported total binding free energies of the ALK-ligand systems were also observed [77].

## 4. Limitations and Improvements in Current Computational Approach

### 4.1. Limitations and Challenges of Current Computational Methods

Current computational approaches utilizing all-atom MD simulations, despite its fruitful aspects, are still limited due to high computational cost. The technological advances and usage of GPU significantly aid in speeding up the sampling [116]. The benchmark in 2011 showed about 10 ns/day using 24 processors of CPU [117], and the benchmark in 2018 shows a significant boost-up of 330 ns/day with a single GPU [116], both applying a 2 fs time step and considering approximately 25,000 atom system. The nature of taking O(*N* log *N*), where N is the number of particles in the system, for particle mesh Ewald (PME) long range interaction computation, however, is unavoidable. Using the state-of-the-art machines and algorithms of unbiased MD, about 2 milliseconds of sampling was needed to generate a reasonable kinetic model to observe associations and dissociations of two proteins showing femtomolar binding affinities [68]. Considering that ordinary experiments trace macroscopic number of proteins with timescales of tens of microseconds to minutes, there is a lot more room for growth for MD simulation that computes single macromolecule for hundreds of nanoseconds to a few microseconds only.

Another concern for MD simulation would be the validity of the result. An ultimate goal of MD would be to yield the same thermodynamic and kinetic observables, which one would find in experiments, but with more atomistic details. To draw this outcome, one would need sufficient amount of sampling from the correct force field. While more precise force fields are being developed, MD users might want to focus on if the right answer, a converged result, has been drawn out from the simulation or not. Evaluating ergodicity from the trajectory and determining whether convergence has been achieved are difficult problems. Sampling complete 3N-dimensional configuration of complex biomolecular system with N particles is simply impossible [118]. When the transitions between states are slow or rare event exists, insufficient sampling will make ensemble averages very sensitive to the number of slow transition or rare events. Even after ensemble averages seem to have converged, one would never know if there is any other unobserved rare event remaining. Many attempts to justify and gauge the quality of MD result have been made [119,120,121,122].

One can argue the convergence of the result using the statistical tools, but it can never be a definitive statement.

### 4.2. Recent Improvements in MD Simulations and Enhanced Sampling Methods

The predictive capacity of MD simulations lies in the reliability of classical potential energy functions, i.e., force fields, to describe the inter- and intramolecular interactions between particles in a system of interest. Force fields encompassed empirically derived energy terms that approximate torsional potentials and interatomic interactions, namely bonded and non-bonded interactions, in a condensed phase. The establishment of a force field that realistically represents the PES of a system is one of the main concerns in MD simulation that developers try to ameliorate through several alterations such as the inclusion of the polarization effect of proteins and solvent, refinement of torsional parameters, and improvement of water models [123,124]. Of particular interest in force field development efforts is the reparameterization of electrostatic interactions (non-bonded) that are governed by the Coulomb’s law equation to characterize interactions between charged atoms [125]. The long-range nature of electrostatic interactions engendered high computational cost. Therefore, to circumvent expensive calculations, traditional force fields have adopted the mean-field treatment of amino acids by assigning fixed partial charges at each atom center. However, the use of fixed point charges overlooked the intrinsic traits of charged atoms to assume lower energy states through the distortion of their electron density cloud and charge redistribution when approached by oppositely charged atoms or in the presence of an external dielectric field. This limitation has been actively rectified over the years through explicit consideration of polarizability, which consequently permitted a more precise electrostatic description of protein atoms in a non-homogeneous natural environment [123,126].

Developers of well-established force fields such as AMBER, AMOEBA. CHARMM, and OPLS customarily utilized three popular methods, *viz.* induced dipole model, the Drude oscillator model, and the fluctuating charge model for straightforward inclusion of electronic polarizability in MD simulations [127,128,129,130,131,132]. The induced dipole model, used in AMBER and AMOEBA polarizable force fields, generally propagates polarizability by assigning inducible dipoles by means of self-consistent field (SCF) calculations based on fixed charges at atom centers, which simultaneously were allocated monopoles. Mutual dipole-monopole interactions as well as dipole-dipole interactions fluctuate with changes in their immediate surrounding electric field, contributing to the additional polarization potential energy term of the force field [130,133,134,135,136,137]. The AMOEBA force field went a step further by considering multipoles through quadrupole moments to reproduce more precisely the anisotropic molecular polarizabilities of non-bonded interactions. On the other hand, the Drude oscillator model, which was established by CHARMM developers, involves the inclusion of a supplementary charged atom attached to atom centers via a harmonic spring. Its displacement from original coordinates when exposed to external electric field mirrors atomic polarizability [128]. The fluctuating charge model, another polarizability model implemented in CHARMM, depicts atomic polarizability by following the principles of electronegativity equalization, whereby charge distribution proceeds until equal chemical potential is achieved between two polarizable cores [129]. Despite differences in the rendition of atomic polarizability among the three methods highlighted (vide supra), a common purpose was to initiate a molecular response towards fluctuations in the surrounding environment during computer simulations, an undertaking that aimed to emulate the intrinsic behavior of particles in nature.

Numerous tinkering has been done to further justify the superiority of polarizable force fields over classical force fields in precisely modelling the physical processes of proteins. Recent improvements in the aforementioned polarizable force fields include the incorporation of screening effects and charge penetration corrections to precisely model the electrostatic energy of short-range interactions, and the consideration of anisotropic polarization of water/solvent molecules to further justify the thesis of polarizability being a many-body effect [127,132,138,139,140]. Consideration of the electrostatic polarization effect has positive implications on numerous protein-related studies [141,142,143,144]. The use of AMOEBA polarizable force field to reproduce the free energy profiles of the permeation of K^+^ and Na^+^ ions through the Gramicidin A channel showed good agreement with experimental observations, in comparison to classical MD simulations [145]. The utilization of Drude polarizable force field has enabled the accurate observation of the mechanism governing the partial activation of a voltage-gated sodium channel. The conformational change of the voltage-sensing domain from resting to the pre-active state and the more precise construction of the free energy profile afforded by the polarizable force field as compared to its non-polarizable counterpart indicated the importance of electrostatic polarization effect in providing a reliable thermodynamic picture of the voltage channel activation [146]. Another recent study exploring the stacking of the aromatic rings of Phe16 and Tyr37 in HIV-1 nucleocapsid protein (NCp7) showed that the explicit treatment of electrostatic polarizability was vital for the accurate reconstruction of the energy profile [147]. Using AMOEBA polarizable force field, the unstacked form of NCp7, characterized by the aromatic rings of Phe16 and Tyr37, was favored and this concurred well with experiment. This is contrary to the simulation performed using a non-polarizable force field, which preferred the stacked conformation—a transient structure according to experiment.

Besides refinement related to polarizability, there are also other improvements, which specifically target certain issues related to current interest in protein research. For instance, the growing focus on PPIs in computational SBDD have unraveled a shortcoming of existing force fields in accurately representing intrinsically disordered proteins (IDPs) or regions (IDRs). IDRs is one of the major contributors towards protein-protein association and current torsion angle potential in existing force fields are not sufficient to precisely depict the flexibility of IDRs. Yu et al. recently developed a non-polarizable, residue-specific force field, called AMBER ff99SBnmr2, to balance the torsional potential to cater to both folded and disordered protein regions. This force field assimilated per-residue dihedral angle modifications, based on experimental information curated from PDB structures of protein fragments (coil library), into the extant ff99SBnmr1—force field that was formulated based on the backbone dihedral potential of ff99SB and optimized using NMR chemical shifts of fully solved proteins [148]. Another recent force field that was developed for protein-protein complexes, and concomitantly achieving optimal description for both folded and disordered regions of a protein, is the *DES-Amber* force field [149]. This force field was developed by reparametrizing dihedral angle parameters, similar to ff99SBnmr2, and optimizing non-bonded interactions through ab initio QM calculations and experimental data. Despite the significant improvement observed for the modelling of protein-protein complexes and individual ordered or disordered proteins, a caveat was added suggesting the necessity of considering polarization effect explicitly for better predictions of protein-protein binding free energies, which in this study showed significant variance from experimental values.

## 5. Prospective Outlook

Recent improvements in MD simulation accompanying the state-of-the-art sampling methods and their applications reveal the robust potential of MD in the field, and the remaining limitations points out where MD needs to be further developed. The unique insights upon the dynamics given by all-atomistic MD simulations are crucial for drug discovery and elucidation of mechanism for many biological processes. MD is an interdisciplinary output from numerous theoretician and experimentalists standing on the fields of physics, chemistry, mathematics, computer science, and many more. As a recent illustration, the burgeoning field of artificial intelligence has now started aiding in boosting the efficiency and accuracy of MD simulation [150]. Machine learning and deep learning techniques have assisted the development of more accurate force fields [151], enhanced sampling through learning algorithms to find optimal reaction coordinates [152], and analyzed data to figure out the underlying free energy surface of the system [153]. The collaborative work among miscellaneous fields has made MD simulation more powerful, and MD will continue benefiting from the growth of affiliated fields. One can run longer simulation with the same amount of real time with the advancements of technology, get a more accurate result with the development of force fields and sampling methods, and tackle various biological problems with recently unveiled crystal structures. However, a careful setup of MD simulations is required to resolve existing biological problems effectively and accurately, and thorough understanding upon components of MD simulations would be beneficial in the long run.
